# Subtle Enhancing Mural Thickening With Diffusion Restriction: An Early MRI Clue to Malignant Transformation in Ovarian Dermoid Cyst

**DOI:** 10.7759/cureus.107465

**Published:** 2026-04-21

**Authors:** Priyanka Priyanka, Gita Devi, Akash Deep

**Affiliations:** 1 Department of Radiology, All India Institute of Medical Sciences, Vijaypur, IND

**Keywords:** dermoid teratoma, malignant transformation, mature cystic ovarian teratoma, ovarian dermoid cyst, squamous cell carcinoma (scc)

## Abstract

Mature cystic teratomas (MCTs) can rarely undergo malignant transformation, most frequently into squamous cell carcinoma. Preoperative diagnosis remains challenging because of the complex internal architecture of dermoid cysts, which may obscure subtle mural malignant components. We report a 46-year-old woman with bilateral ovarian teratomas, in whom malignant transformation within the left ovarian dermoid cyst was not detected on preoperative imaging and was established only on postoperative histopathology. Retrospective review of the preoperative contrast-enhanced magnetic resonance imaging (MRI) demonstrated focal irregular enhancing wall thickening with corresponding diffusion restriction along the cyst wall, correlating with the site of malignant transformation. This case highlights that even subtle enhancing mural thickening with diffusion restriction may represent early malignant transformation, warranting careful evaluation despite the absence of overt invasive features.

## Introduction

Mature cystic teratomas (MCTs), also called dermoid cysts, are the most common benign ovarian tumors, accounting for approximately 20% of ovarian neoplasms [1]. Although mostly benign, 1-2% may undergo malignant transformation, most commonly into squamous cell carcinoma [2-3]. Advanced age and tumor size greater than 10 cm are recognized risk factors [2]. Early and precise preoperative diagnosis is essential for optimal surgical planning and staging [2]. However, differentiating MCTs from those with malignant transformation remains challenging due to the heterogeneous internal composition of dermoid cysts, including fat, keratinaceous material, and calcifications, which may obscure malignant mural components [4].

Although prior studies have reported imaging characteristics of malignant transformation in MCTs, there is a lack of systematic assessment of subtle early MRI features, particularly focal enhancing mural abnormalities. We present a case in which subtle enhancing mural thickening with diffusion restriction on magnetic resonance imaging (MRI) corresponded to the site of malignant transformation on histopathology.

## Case presentation

Clinical presentation

A 46-year-old woman presented with complaints of incomplete voiding of urine for three months and lower abdominal pain for two months. She had a normal obstetric and menstrual history. On clinical examination, a mobile, firm pelvic mass corresponding to 16 weeks' gestational size was palpated. On per speculum examination, the cervix was healthy, pulled up, and displaced anteriorly. On per vaginal examination, bilateral fornix fullness was noted, with a mobile, firm mass palpable in the pouch of Douglas.

Investigations

Blood investigations revealed Hb of 11.8 g/dL, total leukocyte count of 5,000/µL, platelet count of 190,000/µL, and thyroid-stimulating hormone (TSH) of 4.17 mIU/L. Liver and renal function tests were normal. Serology was nonreactive. Urine routine and microscopy were normal. The Pap smear was negative for intraepithelial lesion or malignancy, and cancer antigen-125 (CA-125) levels were elevated (185.90 U/mL) (Table 1).

**Table 1:** Preoperative laboratory investigations

Laboratory tests	Value	Reference range
Hemoglobin	11.8 g/dL	12-16 g/dL (female)
Total leukocyte count	5,000/µL	4,000-11,000/µL
Platelet count	190,000/µL	150,000-450,000/µL
Thyroid-stimulating hormone	4.17 mIU/L	0.4-5.0 mIU/L
Liver function test	Within normal limits	Within normal limits
Renal function test	Within normal limits	Within normal limits
Serology	Non-reactive	Nonreactive
Urine routine and microscopy	Within normal limits	Within normal limits
Pap smear	Negative for intraepithelial lesion or malignancy	Negative for intraepithelial lesion or malignancy
Cancer antigen-125	185.90 U/mL	0-35 U/mL

Imaging findings

**Figure 1:**
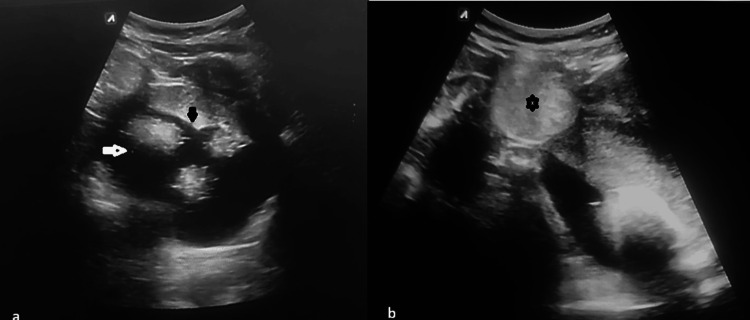
Ultrasonography images (a) Ultrasonography of the largest left ovarian cystic lesion demonstrating hyperechoic floating spherules (white arrow) and a fat-fluid level (black arrow). (b) Ultrasonography of a small right ovarian lesion demonstrating diffuse hyperechogenicity (asterisk)

Ultrasonography revealed a large heteroechoic cystic lesion in the left ovary measuring 11 × 10.4 cm with internal hyperechoic contents, floating hyperechoic spherules, fat-fluid level, and dot-dash appearance without posterior acoustic shadowing. A well-defined heterogeneous hyperechoic cystic lesion was also noted in the right ovary, measuring 5.1 × 4.5 cm (Figure 1). No definite solid mural nodule or papillary projection was identified. On color Doppler evaluation, no internal vascularity was demonstrated within the lesion. Based on the imaging findings, an initial diagnosis of a benign MCT (dermoid cyst) was made.

MRI demonstrated multiple bilateral cystic lesions containing fat, with two in the left ovary and one in the right ovary. The largest lesion, located in the left ovary, measured 12.4 × 10.8 cm. T1-weighted imaging with and without fat saturation demonstrated characteristic hyperintense fatty components, including a Rokitansky nodule along the left posterolateral cyst wall, floating spherules, and a fat-fluid level. Diffusion-weighted imaging showed focal restriction along the left anterolateral cyst wall, as well as within the spherules and at the fat-fluid interface, with additional subtle diffusion restriction along the right lateral cyst wall. Postcontrast T1 fat-suppressed images demonstrated thin smooth cyst wall enhancement, residual enhancing ovarian parenchyma clawing the anterior surface of the cyst, and no discernible enhancement within the Rokitansky nodule (Table 2). No extramural invasion, ascites, or lymphadenopathy was identified.

**Table 2:** MRI features of benign and malignant components in ovarian mature cystic teratoma MRI: magnetic resonance imaging

Imaging features within the largest ovarian cyst	Diffusion restriction	Postcontrast enhancement	Diffusion restriction and enhancement
Floating spherules	Mild	-	-
Fat-fluid level	Mild	-	-
Rokitansky nodule	-	-	-
Cyst wall	Right lateral wall (mild)	Thin smooth peripheral wall enhancement. Ovarian parenchyma clawing (anteriorly)	Irregular thickening (left anterolateral wall)
Additional left ovarian cyst	Diffuse central restriction	-	–

**Figure 2:**
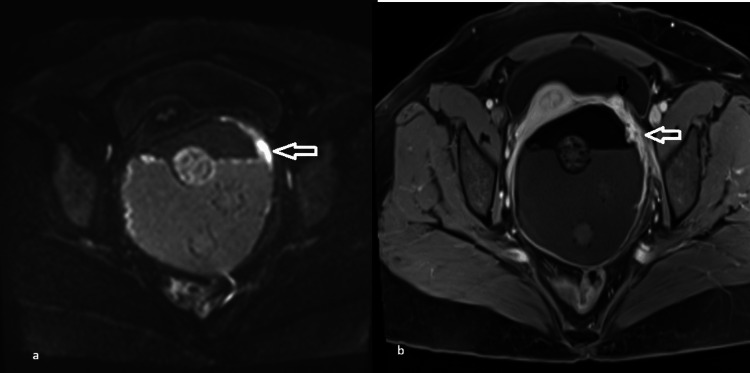
Enhancing mural thickening with diffusion restriction on MRI MRI: magnetic resonance imaging (a) Diffusion-weighted image (b = 1400 s/mm²) demonstrating focal mural thickening along the cyst wall with corresponding hyperintense signal indicating restricted diffusion (white arrow). (b) Axial T1-weighted fat-saturated postcontrast image showing subtle enhancing wall thickening (white arrow) along the cyst wall, with adjacent residual enhancing ovarian parenchyma (black arrow)

**Figure 3:**
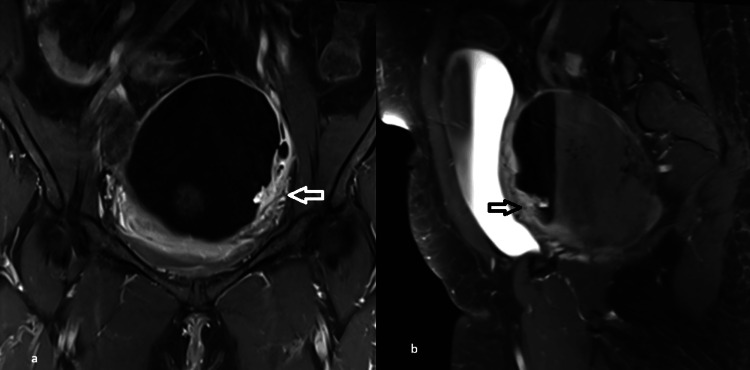
Multiplanar MRI revealing irregular enhancing mural thickening MRI: magnetic resonance imaging (a, b) Coronal and sagittal T1-weighted fat-saturated postcontrast images demonstrating irregular enhancing mural thickening along the cyst wall (arrows), better appreciated on multiplanar imaging

On retrospective review, a focal irregular enhancing endophytic sessile area of wall thickening (maximum thickness: 7.3 mm) was identified along the left anterolateral cyst wall on postcontrast T1 fat-suppressed images, with corresponding diffusion restriction (ADC 1.1 x 10⁻³ mm²/s), raising suspicion for malignant transformation (Figures 2-3).

Treatment and outcome

**Figure 4:**
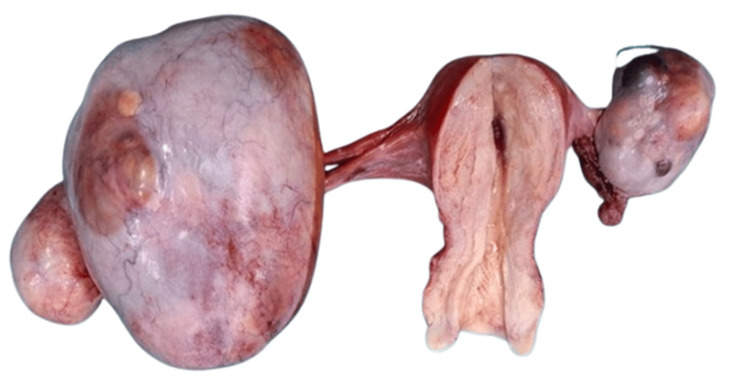
Gross specimen Gross specimen showing the uterus (central cut section) with bilateral ovarian cystic lesions demonstrating smooth external surfaces and intact capsules

The patient underwent transabdominal hysterectomy with bilateral salpingo-oophorectomy. The postoperative specimen revealed a bulky uterus, enlarged left ovary containing two smooth encapsulated cysts, and enlarged right ovary with a multilocular cyst (Figure 4). No ascites or pelvic lymphadenopathy was found intraoperatively. No peritoneal washings, omental biopsies, or lymph node dissections were performed. On follow-up imaging, positron emission tomography (PET) and contrast-enhanced computed tomography (CECT) scans revealed no residual or metastatic lesions. The patient received six cycles of platinum-based chemotherapy and remains disease-free at one-year follow-up.

Histopathology

**Figure 5:**
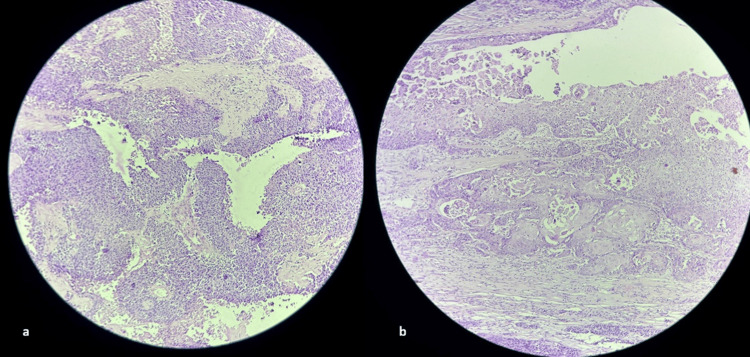
Squamous cell carcinoma Histopathological examination (hematoxylin and eosin stain) showing nests of moderately differentiated squamous cell carcinoma infiltrating the stromal tissue

Histopathology revealed features of a left ovarian MCT with a thickened area in its left lateral cyst wall, showing moderately differentiated squamous cell carcinoma (Figure 5). Foci of transitional-like epithelium with urothelial differentiation were also identified. The capsule was intact, and no ovarian surface involvement was noted. The right ovary showed histological features of an MCT. The uterine myometrium revealed adenomyosis.

## Discussion

While malignant transformation in MCTs occurs in only 1-2% of cases, prompt recognition is essential as it affects patient management and prognosis. Tumor stage at the time of diagnosis is the most important prognostic factor [5]. Although advanced age (generally after 40 years), postmenopausal status, large tumor size (>10 cm), and elevated CA-125 are recognized risk factors [6], imaging findings often remain subtle. In this patient, the malignant focus was not identified preoperatively, highlighting limitations in current diagnostic paradigms. Knowledge of subtle imaging features of malignant transformation can prevent the misdiagnosis of early-stage disease and improve patient survival.

Imaging features suggestive of malignant transformation include a solid mural nodule or papillary projection, irregular wall thickening, irregular tumor margins, extracapsular invasion into adjacent organs, peritoneal dissemination, lymphadenopathy, and ascites [7-9]. On ultrasonography, the presence of a solid component with internal vascularity on color Doppler is particularly concerning [10]. In our case, the key finding was focal, irregular, enhancing wall thickening with an endophytic, sessile configuration. Such a pattern has been described in association with malignant transformation in previous studies by Kawaguchi et al. [9]. However, this finding is not specific and should be interpreted in conjunction with other imaging features. Unlike typical cases with a dominant solid component, our case demonstrated only subtle mural thickening without overt invasive features, making detection particularly challenging. Additionally, the presence of residual enhancing ovarian parenchyma adjacent to the area of mural thickening further obscured the subtle malignant spot. 

Ovarian MCTs frequently show internal solid components with fatty tissue, ringlike enhancement, and a low-risk time-intensity curve on dynamic contrast-enhanced MRI [11]. The distinction between benign and malignant enhancing components is important. 

Diffusion restriction in malignant transformation reflects increased cellularity and reduced extracellular space [12]. However, it is not specific, as benign dermoid cysts may also show restriction due to keratinous contents [13]. Although diffusion restriction was noted within keratinous components such as spherules and fat-fluid interfaces, these did not show corresponding enhancement. In this case, only the malignant area demonstrated both enhancement and diffusion restriction. Therefore, correlation with postcontrast imaging is essential. Another observation was that the lesion was more conspicuous on coronal and sagittal imaging, highlighting the importance of careful evaluation in multiple planes. This case highlights the importance of careful evaluation of cyst walls in multiple planes and correlation of diffusion-restricting areas with contrast enhancement, especially in high-risk patients.

## Conclusions

Malignant transformation of MCT is rare and often difficult to diagnose preoperatively. Radiologists should maintain a high index of suspicion in patients with established risk factors and correlate enhancing mural thickening with diffusion restriction, as diffusion restriction alone may be misleading in dermoid cysts. Multiplanar evaluation is crucial, as subtle malignant foci may be inconspicuous on axial images but more readily appreciated on coronal and sagittal images.
